# POMONA: a multiplatform software for modeling seed physiology

**DOI:** 10.3389/fpls.2023.1151911

**Published:** 2023-07-06

**Authors:** Renato Fernandes Cantão, João Paulo Ribeiro-Oliveira, Edvaldo A. Amaral da Silva, Amanda Rithieli dos Santos, Rute Quelvia de Faria, Maria Marcia Pereira Sartori

**Affiliations:** ^1^ Center for Science and Technology for Sustainability (CSTS), Federal University of São Carlos (UFSCar), Sorocaba, SP, Brazil; ^2^ Instituto de Ciências Agrárias (ICIAG), Universidade Federal de Uberlândia (UFU), Uberlândia, Minas Gerais, Brazil; ^3^ Department of Crop Science, College of Agricultural Sciences, São Paulo State University (UNESP), Botucatu, SP, Brazil

**Keywords:** agriculture 5.0, algebraic models, automated scoring, corn, seed quality, soybean, plant physiology

## Abstract

Seed physiology is related to functional and metabolic traits of the seed-seedling transition. In this sense, modeling the kinetics, uniformity and capacity of a seed sample plays a central role in designing strategies for trade, food, and environmental security. Thus, POMONA is presented as an easy-to-use multiplatform software designed to bring several logistic and linearized models into a single package, allowing for convenient and fast assessment of seed germination and or longevity, even if the data has a non-Normal distribution. POMONA is implemented in JavaScript using the Quasar framework and can run in the Microsoft Windows operating system, GNU/Linux, and Android-powered mobile hardware or on a web server as a service. The capabilities of POMONA are showcased through a series of examples with diaspores of corn and soybean, evidencing its robustness, accuracy, and performance. POMONA can be the first step for the creation of an automatic multiplatform that will benefit laboratory users, including those focused on image analysis.

## 1 Introduction

The perspective of automation in data collection and analysis is feasible and offers an opportunity to improve inter- and intra-laboratory quality control in science (Benos et al., 2021; Rejeb et al., 2022). For seed science, this quality not only guarantees reproducibility and replicability of results, but also plays a central role for national and international trade (Katalin et al., 2009). Taking this into account, robust algorithms, using artificial intelligence and machine learning, have been considered the future for quality operations, specially by integrating image analysis and processing with classical physiological measurements (Deal et al., 2020; de Medeiros et al., 2020; Galletti et al., 2020; Ribeiro-Oliveira et al., 2020; Barboza da Silva et al., 2021; Batista et al., 2022; Oliveira et al., 2022). This, for example, can also be observed in platforms for phenotype analysis during the seed-seedling transition, such as SeedGerm (Colmer et al., 2020), or ScreenSeed, a novel high throughput seed germination phenotyping method based on computer vision (Merieux et al., 2021). It is possible to mention other examples of this technology transference such as the GERMINATOR, a high throughput scoring and curve fitting software for seed germination (Joosen et al., 2010; Ligterink and Hilhorst, 2017), and the SeedStor, a publicly available database for the seed collections held by the Germplasm Resources Unit (GRU) (Horler et al., 2018), It’s important to highlight that other phenotyping high throughput systems have been proposed over the years, including a chlorophyll fluorescence-based imaging (ChIF) system to detect emerging cotyledons (Pavicic et al., 2019), and an automatic computer vision system using RGB image-based analysis to detect radicle emergence (Ducournau et al., 2005). The next step is to use an interface such as the web that would not only be used as a way to transition traditional methods to a fully automated system, but also be an important extension tool. This would make aspects that are little explored more available and promote the development of seed science. That’s inspired the development of POMONA. This is the name of the Roman goddess of fruit trees, gardens, and orchards.

One of the biggest bottlenecks for seed scientists is to manage the multidisciplinary aspects that the area presents. Over the last century, for example, several authors have promoted seed germination and seedling emergence modeling tools in order to make the process highly elucidated (Ranal and de Santana, 2006; Mcnair et al., 2012). However, few scientists were able to absorb this technology, since algebraic calculations did not always make the measurements accessible (Ranal et al., 2009). Specifically, in the last twenty years many advances have occurred in the standardization and use of measurements of kinetics, uniformity and synchrony of germination (Ribeiro-Oliveira and Ranal, 2014; 2016). This was possible through the popularization of reviews on the subject and papers specifically aimed at calculating these measurements, which go from the preparation of spreadsheets (Ranal et al., 2009) to insertions of packages still incipient in recent programming languages, such as R project (Lozano-Isla et al., 2019; Silva et al., 2019). Regardless of the strategy, the volume of users of the measurements and the clarifications given by them to physiological phenomena led to improvements in aspects of biology, physiology, ecology and seed technology. Nevertheless, there is still a lack of technological interfaces that provide a bridge between academia and seed laboratories, such as an free and easy-to-use web interface, with the ability to promote insights on important aspects, such as viability, longevity and vigor.

Currently, the proposal of Joosen et al. (2010) and Silva et al. (2019) present some solutions for the calculation of longevity, but articulations, especially that respect the statistical context of non-normality, still need to be introduced. Therefore, robust models, using Generalized Linear Models, were proposed, reducing the need of statistical knowledge (dos Santos et al., 2019; de Faria et al., 2020). This is an important perspective, especially for the calculation of parameters sensitive to deviations to the Normal distribution, such as P_50_. The calculations for P_50_, classically used by seed scientists to determine seed longevity (presenting central role in germplasm banks) and to promote insights on the physiology of vigor samples, are still restrictively performed due to the poor familiarity of technicians and scientists of the area with statistics. Taking this into account, POMONA is presented as a technological resource for seed scientists and technologists. The intention is not only to promote a transition from traditional systems to more robust features such as current algorithms, but also to promote an interactive tool for calculating parameters associated with vigor in order to contextualize new insights from the area, enabling easier decision making. That is the first step to introduce image analysis to technical laboratories. The aim, therefore, is to introduce a new processing tool, which will promote the inclusion of insights from the physiological/experimental statistics interface.

## 2 Methodology

Two biological models were used to prove the applicability and robustness of POMONA. They were soybean (*Glycine max* L.) seeds and corn (*Zea mays* L.) caryopses (henceforth called seeds for technical purposes). The choice of these biological models was because soybean and corn are the most cultivated eudicotyledonous and monocotyledonous species around the world, respectively (USDA, 2022). Consequently, those species also are routinely analyzed by seed technologists worldwide.

### 2.1 Seed production and physiology

Seeds were collected from commercial trials around Brazil (South, Midwest and North regions) crop season of 2017/2018. In a pre-test, we selected twenty-three seed lots from commercial soybean cultivars and six seed lots from commercial corn cultivars as a study case to apply POMONA. In general, seed samples had approximately 5-7 kg both for soybean and corn. As they were part of a commercial field, the seeds were mechanically harvested. They were then placed in paper bags and stored at 10°C/55% relative humidity (RH) until the beginning of the experiment, which was no more than 30 days later.

The water content of the soybean and corn seeds was determined by oven drying at 105 ± 3°C for 24 hours with five replicates of 20 seeds (*n* = 100) and expressed in percentage. Soybean seedlings in post-germination (i.e., normal seedlings) were evaluated by using six replicates of 50 seeds using germination paper, imbibed in water at 25 °C in the dark in biochemical oxygen demand (BOD) chambers. The first count of normal seedlings, a vigor test according to ISTA (2019), was performed 5 days after sowing (DAS), and the final count was done 8 DAS.

Two classical tests were also used for analysis of seedling vigor: 1. accelerated aging for soybean; and 2. cold test for corn. These tests are the most used for vigor classification in seed lots for each of the species analyzed here. The accelerated aging test was performed on soybean seeds during 24 hours at 95% RH at 41°C. For corn seeds, the post germination test was done with six replicates of 50 seeds sown in germination paper imbibed in water at 25°C under constant light in BOD chambers. The first normal seedling count was performed 4 DAS, and the final count was done 7 DAS. All the results were expressed in terms of percentage of normal seedlings. The cold test without soil was conducted with four replicates of 50 seeds, on paper towels moistened with water at 2.5 times the dry paper weight. The rolls were packed in plastic bags and kept in a cold chamber at 10°C for seven days. After that, they were taken out of the plastic bags and placed in a germinator at 25°C, under constant light, for four days (Barros et al., 1999). The percentage of normal seedlings was calculated. The longevity of soybean and corn seeds was assessed by keeping the seeds at constant 50%, 60%, 75% and 80% of RH at 35°C in hermetically sealed plastic boxes. At different times during storage, seeds were imbibed as described above and germination was recorded. In addition, median time for seed germination was expressed by t_50_, defined by the time that 50% of the seeds took to germinate. Longevity was expressed by P_50_, defined as the time (days) after which the seeds lost 50% of their viability during storage.

In parallel to the seedling assays, the germination process was evaluated by considering seeds as germinated when the protruded radicle showed a length of 2 mm. The records were processed daily at a same hour. From these protrusion data, germinability (G, Labouriau, 1983); mean germination time ( $\bar{t}$ , Labouriau, 1983); median germination time (t_50_, Coolbear et al., 1984) and mean germination rate ( $\bar{v}$ , Labouriau, 1970) were calculated. In addition, time between 16% and 84% of germinated seeds (u_8416_), area under curve before 120 h (AUC_120_) and time for 10% of seed sample germination (t_10_) were calculated according to Joosen et al. (2010). Student’s *t* test (at 0.05 significance) was used for comparison between samples for each of these measurements.

### 2.2 Model fitting

The curves from the longevity, germination or seedling in post-germination versus time were plotted. To predict the P_50_ time, the following models were used: a. $F\left(p\right)={\text{Φ}}^{-1}\left(p\right)$ (probit; Ellis and Roberts, 1980), and b. F(p)=ln[p/(1−p)]  (logit; Faria et al., 2020). The models of Boltzmann, Gompertz and Hill were analyzed with times from t = 0 to t = m, where m differs depending on the species. The Boltzmann model can be defined as: $y=\frac{A_{1}-A_{2}}{1+e^{\left(t-t_{0}\right)/dt}}+A_{2}$ , where: A_1_ = Initial frequency; A_2_ = Final frequency; dt = time constant; t = experimental time; t_0_ = time in which the frequency is 50% (t_(1/2)_); e = base of the neperian logarithm. In this function, the value of t_50_ is directly determined by the fit. The Gompertz model can be defined by $y=ae^{-e^{(-k\left(t-tc\right)}}$ , where: y= accumulated percentage of germination or post germination at time t; a = the maximum percentage of accumulated germination or post germination; k = the germination or post germination velocity to reach the asymptotic value; t = time; tc = time at which y reaches half of its maximum value; e = base of the neperian logarithm. The Hill model by El-Kassaby et al. (2008) is $y=y0+ax^{b}/\left(c^{b}+x^{b}\right)$ , where: y is the accumulated percentage of germination or post germination at time t, y0  is the intercept in the y axis, a is the maximum percentage of accumulated germination or post germination, b is the parameter to control the behavior curve, and c  is the t_50_. The data were evaluated for normality before and after data transformation according to the Kolmogorov-Smirnov test (at 0.05 significance).

#### 2.2.1 Adjustment quality predictors

The model fitting was determined according to biological significance and: a. The residual standard deviation was ${\text{SD}}_{R}=\sqrt{MSE/\left(n-p\right)}$ , where MSE = mean square error, n = number of observations and p = number of model parameters, the smaller the value of the residual standard deviation, the better the fit of the model. b. The adjusted coefficient of determination was (R^2^ adj.) $R^{2}adj=1-\left[\frac{\left(1-R^{2}\right)\left(n-1\right)}{n-p}\right]$ : where $R^{2}=1-\frac{SSR}{SST}$ is the coefficient of determination, SSR is sum square regression, SST is sum square total; p is the number of model parameters; n is number of observations.

### 2.3 The hardware of POMONA

POMONA was developed entirely in JavaScript (ECMA International, 2017), using the asynchronous execution environment Node.js, version 14.16.1 (https://nodejs.org/en/). POMONA can import data directly from Microsoft Excel (tm) spreadsheets, using SheetJS, version 0.17.4 (https://github.com/SheetJS/sheetjs), and CSV files (*comma-separated values*), using PapaParse version 5.2.0 (https://www.papaparse.com).

The data processing is comprised of in-house implementations of some basic vector algebra functions (sum, subtraction, dot product, norm) used in the construction of logistic and linear models. These were fitted by a version of the Levenberg-Marquardt method for nonlinear least squares approximation, using the ml-levenberg-marquardt library, version 3.1.1 (https://github.com/mljs/levenberg-marquardt). It provides a set of parameters for fine tuning the method, including the damping and the error tolerance for the numerical approximation of the Jacobian matrix.

POMONA’s validation (both germination/vigor and longevity) was performed by fitting a logistic model with probit and logit link functions. By using this combination, P_50_ and t_50_ values were estimated as the central point for viability and longevity analyses, respectively. For viability, the direct association was performed between Hill’s model inferences, estimated by GERMINATOR, and inferences from a logistic model with link functions (probit or logit). The robustness of our software was proved by an overlapping between confidence intervals (α = 0.05) calculated for POMONA parameter models.

## 3 Results

### 3.1 Biological models: description and validation from a physiological and technological perspective

In general, freshly collected (both corn and soybean) seeds had high viability, demonstrating germinability higher than 80% (**Tables 1**, **2**). Two exceptions for soybean cultivar samples (*G* = 73.75%) and one for hybrid corn (*G* = 79%) were observed. Four germinability patterns described the soybean cultivar samples (**Table 1**), and two patterns demonstrated the hybrid corn behavior (**Table 2**). On the other hand, the measurements of kinetics, time and uniformity, as well as the AUC index, demonstrated more physiological differences among the samples (**Tables 1**, **2**). In this case, two samples were outliers for soybean, SAM07 and SAM22. Seeds from SAM07 showed a more rapid process (see $\bar{v}$ ; **Table 1**), possessing an earlier (t_10_; **Table 1**) and regular (u_8416_; **Table 1**) germination, which gave them a precocious pattern (t_50_ and $\bar{t}$ ; **Table 1**) for event peaks in the sample. Consequently, this sample presented the highest germinability associated with a first germination peak and a more regular process (see AUC; **Table 1**). However, SAM22 showed an inverse pattern for all these measurements (**Table 1**).

**Table 1:** Germination process of soybean (*Glynice max* L.) seeds from different cultivars used as biological model for POMONA’s validation.

Cultivar Sample^1^	Functional traits^2,#^						
	*G* (%)	t_50_ (h)	t_10_ (h)	$\bar{t}\;$ (h)	$\bar{v}$ (h^-1^)	u_8416_ (h)	AUC_120_ (units)
**SAM14**	100 a	34.75 b	22.79 b	31.36 b	0.03199 b	14.47 c	88.99 a
**SAM08**	100 a	49.06 c	35.51 d	51.03 f	0.02015	24.89 f	86.29 b
**SAM07**	100 a	26.17 a	19.65 a	26.05 a	0.03865 a	11.37 a	91.30 a
**SAM13**	100 a	31.90 b	25.60 c	31.61 b	0.03187 b	10.89 a	86.29 b
**SAM17**	100 a	50.29 c	41.71 e	50.57 f	0.02013 e	14.37 c	69.10 d
**SAM21**	100 a	34.82 b	24.74 b	35.70 c	0.02839 c	18.08 d	81.32 c
**SAM01**	100 a	33.10 b	26.19 c	33.11 b	0.03172 b	11.80 b	86.29 b
**SAM02**	100 a	34.82 b	27.68 c	34.84 c	0.02893 c	12.15 b	84.36 b
**SAM18**	100 a	40.40 b	35.07 d	39.85 d	0.02534 d	8.65 a	79.34 c
**SAM06**	98.75 a	46.53 c	34.61 d	47.69 e	0.02129 e	21.01 e	71.24 d
**SAM11**	98.75 a	36.09 b	27.49 c	36.41 c	0.02919 c	14.78 c	69.09 d
**SAM12**	97.50 a	51.96 c	40.81 e	52.61 f	0.01926 e	19.06 d	65.12 e
**SAM09**	95.00 b	36.38 b	28.43 c	36.26 c	0.03113 b	13.31 b	78.75 c
**SAM19**	95.99 b	49.08 c	40.23 e	48.80 e	0.02075 e	14.26 c	66.27 e
**SAM00**	93.75 b	50.00 c	34.80 d	48.86 e	0.02144 e	24.43 f	67.48 e
**SAM05**	93.75 b	34.75 b	26.96 c	34.34 c	0.02947 c	12.78 b	79.03 c
**SAM16**	92.50 b	33.66 b	23.17 b	33.56 b	0.02997 c	17.71 d	78.13 c
**SAM10**	92.50 b	26.51 a	20.48 a	25.79 a	0.03890 a	9.89 a	86.02 b
**SAM15**	92.50 b	32.13 b	24.62 b	31.60 b	0.03256 b	12.34 b	79.91 c
**SAM20**	86.25 c	47.51 c	34.11 d	46.42 e	0.02182 e	21.57 e	61.47 f
**SAM22**	83.75 c	125.22 d	91.00 f	119.44 g	0.00908 f	58.09 g	15.23 g
**SAM03**	73.75 d	46.47 c	29.99 c	39.94 d	0.02550 d	18.63 d	57.19 f
**SAM04**	73.75 d	40.83 c	28.07 c	35.83 c	0.02818 c	15.26 c	60.37 f

^1^: Soybean sample code for commercial lots used as biological model; ^2^: Germination measurements used to analyze seed physiology; G: Germinability; t_50_: median germination time, t_10_: time to 10% of seeds in the sample are germinated; $\bar{t}$ : mean germination time; $\bar{v}$ : mean germination rate; u_8416_: time between 16% and 84% of germinated seeds**;** AUC_120_: area under the curve until 120 h.^#^: For each functional trait, the values followed by the same letter (in column) do not differ by the Student t-test (α = 0:05).

**Table 2:** Germination process of corn (*Zea mays* L.) seeds from different cultivars used as biological model for POMONA’s validation.

Hybrid Sample^1^	Functional traits^2,#^						
	*G* (%)	t_50_ (h)	t_10_ (h)	$\bar{t}\;$ (h)	$\bar{v}$ (h^-1^)	u_8416_ (h)	AUC_120_ (units)
**MAM01**	79 b	53.8 d	41.9 b	51.1 c	0.0196 b	16.4 c	53.8 c
**MAM03**	99 a	52.7 c	40.0 b	53.4 d	0.0187 b	21.5 d	64.0 b
**MAM04**	94 a	53.4 d	45.2 c	53.1 d	0.0188 b	13.2 b	62.3 b
**MAM05**	99 a	40.6 a	33.1 a	40.5 a	0.0251 a	12.5 a	77.9 a
**MAM07**	93 a	48.1 b	40.1 b	47.9 b	0.0219 a	13.1 b	66.9 b
**MAM01**	79 b	53.8 d	41.9 b	51.1 c	0.0196 b	16.4 c	53.8 c

^1^: Corn sample code for commercial lots used as biological model; ^2^: Germination measurements used to analyze seed physiology; G: Germinability; t_50_: median germination time, t_10_: time to 10% of seeds in the sample are germinated; $\bar{t}$ : mean germination time; $\bar{v}$ : mean germination rate; u_8416_: time between 16% and 84% of germinated seeds**;** AUC_120_: area under the curve until 120 h.^#^: For each functional trait, the values followed by the same letter (in column) do not differ by the Student t-test (α = 0:05).

For corn seeds, MAM 01 and MAM 05 stood out with low and high physiological quality, respectively (**Table 2**). This biological model was effective to demonstrate that seeds with high capacity to germinate have different behaviors regarding kinetics. This can affect time for early events and, consequently, peak germination over time (see t_10_, t_50_ and $\bar{t}$ in MAM04 and MAM07; **Table 2**). However, the regularity in which these events occurred in the early steps of germination is what defines how early the seed-seedling transition occurs (see u_8416_ in MAM03 and MAM04 or MAM05 and MAM07; **Table 2**). A consequence is that samples which take longer to reach germination peak due to slower kinetics had a similar AUC index to the others that were more precocious (see MAM04 and MAM07; **Table 2**). These behaviors validated both biological models to observe the applicability of POMONA from a physiological point of view.

The samples of soybean and corn seeds had high capacity to germinate after 30 days (see embryo protrusion in **Tables 3**, **4**). However, a high variation among the samples analyzed (CV SMA: 73.8%; CV MAM: 89.5%) refuted the pre-testing in which germination for both species had several patterns for seed germination. For soybean seeds, three germination patterns were observed (low: 73.8% ≤ *G* ≤ 78.5%; intermediate: 86.5% ≤ *G* ≤ 87.0%; high: 91.5% ≤ *G* ≤ 100%). The corn seed samples demonstrated two germination patterns and only one hybrid sample had low germinability (*G* = 67.5% **Table 3**).

**Table 3:** Seed-seedling transition in soybean (*Glycine max* L.) seeds of different cultivar samples after 30 days collected and from a technological point of view.

Cultivar Sample^1^	Technological traits^2,#^				
	*G* (%)	*NS* (%)	*FC* (%)	AA (%)	WC (%)
**SAM17**	100.0 a	92.5 a	0.0 e	62.0 d	6.8 a
**SAM21**	100.0 a	69.5 c	25.6 c	95.5 a	11.0 b
**SAM02**	99.0 a	94.0 a	72.5 b	82.5 b	7.6 a
**SAM08**	99.0 a	87.0 b	70.5 b	71.5 c	7.5 a
**SAM14**	98.5 a	89.5 b	76.0 b	65.0 d	7.7 a
**SAM20**	97.5 a	93.5 a	6.0 e	37.1 e	7.2 a
**SAM07**	97.5 a	88.5 b	73.0 b	93.5 a	7.9 a
**SAM01**	97.0 a	91.0 a	17.0 e	86.0 b	7.7 a
**SAM19**	96.4 a	79.3 b	39.44 d	49.9 d	7.2 a
**SAM13**	96.0 a	89.5 b	86.5 a	72.0 c	7.6 a
**SAM00**	94.0 a	93.5 a	86.0 a	71.0 c	7.1 a
**SAM11**	94.0 a	91.0 a	87.0 a	76.0 c	7.6 a
**SAM05**	94.0 a	88.0 b	83.5 a	49.0 d	7.4 a
**SAM12**	93.5 a	85.5 b	79.5 a	79.4 b	7.6 a
**SAM22**	93.5 a	77.5 c	6.0 e	12.0 e	8.2 a
**SAM06**	93.0 a	85.5 b	77.5 b	50.5 d	7.7 a
**SAM18**	92.4 a	90.4 a	77.4 b	90.0 a	7.3 a
**SAM09**	91.5 a	81.7 b	62.9 c	59.5 d	7.4 a
**SAM10**	89.6 b	79.7 b	71.8 b	65.5 d	7.4 a
**SAM16**	87.0 b	48.0 d	41.5 d	20.0 e	7.6 a
**SAM15**	86.5 b	63.0 c	49.0 d	24.5 e	7.6 a
**SAM04**	78.5 c	60.5 c	43.5 d	0.0 e	7.7 a
**SAM03**	73.8 c	56.7 c	38.2 d	0.0 e	7.8 a

^1^: Soybean sample code for commercial lots used as biological model; ^2^: Measurements used to analyze seed technology; G: Germinability; NS: Normal Seedlings developed in the sample; FC: Normal Seedlings developed in the sample at first count of seedling development test; AA: Normal seedlings developed after accelerated aging; WC: Water content of seeds in anydrobiosis.^#^: For each functional trait, the values followed by the same letter (in column) do not differ by the Student t-test (α = 0:05).

**Table 4:** Seed-seedling transition in corn (*Zea mays* L.) seeds of different hybrid samples after 30 days collected and from a technological point of view.

Hybrid Sample^1^	Technological traits^2,#^				
	*G* (%)	*NS* (%)	*FC* (%)	AA (%)	WC (%)
**MAM03**	98.5 a	87.5 a	50.5 b	70.5 b	11.7 a
**MAM06**	97.5 a	88.5 a	81.0 a	82.0 a	10.1 a
**MAM05**	94.0 a	90.5 a	88.0 a	85.5 a	11.3 a
**MAM01**	90.5 a	83.5 a	40.0 b	71.4 b	11.2 a
**MAM04**	89.0 a	63.5 b	15.5 c	67.5 b	12.4 a
**MAM02**	67.5 b	35.0 c	20.0 c	32.4 c	10.4 a

^1^: Corn sample code for commercial lots used as biological model; ^2^: Measurements used to analyze seed technology; G: Germinability; NS: Normal Seedlings developed in the sample; FC: Normal Seedlings developed in the sample at first count of seedling development test; AA: Normal seedlings developed after accelerated aging; WC: Water content of seeds in anydrobiosis.^#^: For each functional trait, the values followed by the same letter (in column) do not differ by the Student t-test (α = 0:05).

On the other hand, normal seedlings in immediate post-germination were segregated into three groups both for soybean and corn samples (**Tables 3**, **4**). For soybean seeds the patterns (low transition: 48.0% ≤ *NS* ≤ 63.0%; intermediate transition: 69.5% ≤ *NS* ≤ 79.7%; high transition: 85.5% ≤ *NS* ≤ 100%) showed minor variation between samples (CV: 17.24%) compared to the corn seed patterns (CV: 74.7%; low transition: *NS* = 35%; intermediate transition: *NS* = 63.5%; high transition: 83.5% ≤ *NS* ≤ 90.5%).

It is important to note that the seed-seedling transition for samples of both species had different patterns in relation to the expected from those observed previously in seed germination, i.e., there were samples with high germinability but an intermediate pattern for normal seedling development (for example, SAM21; MAM04). That validated biological models, since segregation was related to seedling vigor (**Tables 3**, **4**), a common use for P_50_ and t_50_ analysis. In general, the sample presented different vigor behavior when analyzed by first count (FC) and accelerated aging (AA) or cold test (CT) (**Tables 3**, **4**). Despite this, three groups of samples were determined by the two vigor tests (FC and AA for soybean; FC and CT for corn). In addition, corn seeds demonstrated a similar initial water content (10.1% ≤ *WC* ≤ 12.4%), whereas soybean seeds had only one sample outlier (*WC* = 11.0%). The other soybean samples were similar in this characteristic (6.8% ≤ *WC* ≤ 8.2%; **Tables 1**, **2**). Therefore, these behaviors also validated biological models to analyze POMONA’s applicability from a technological point of view.

### 3.2 POMONA’s interface and layout

**Figure 1:**
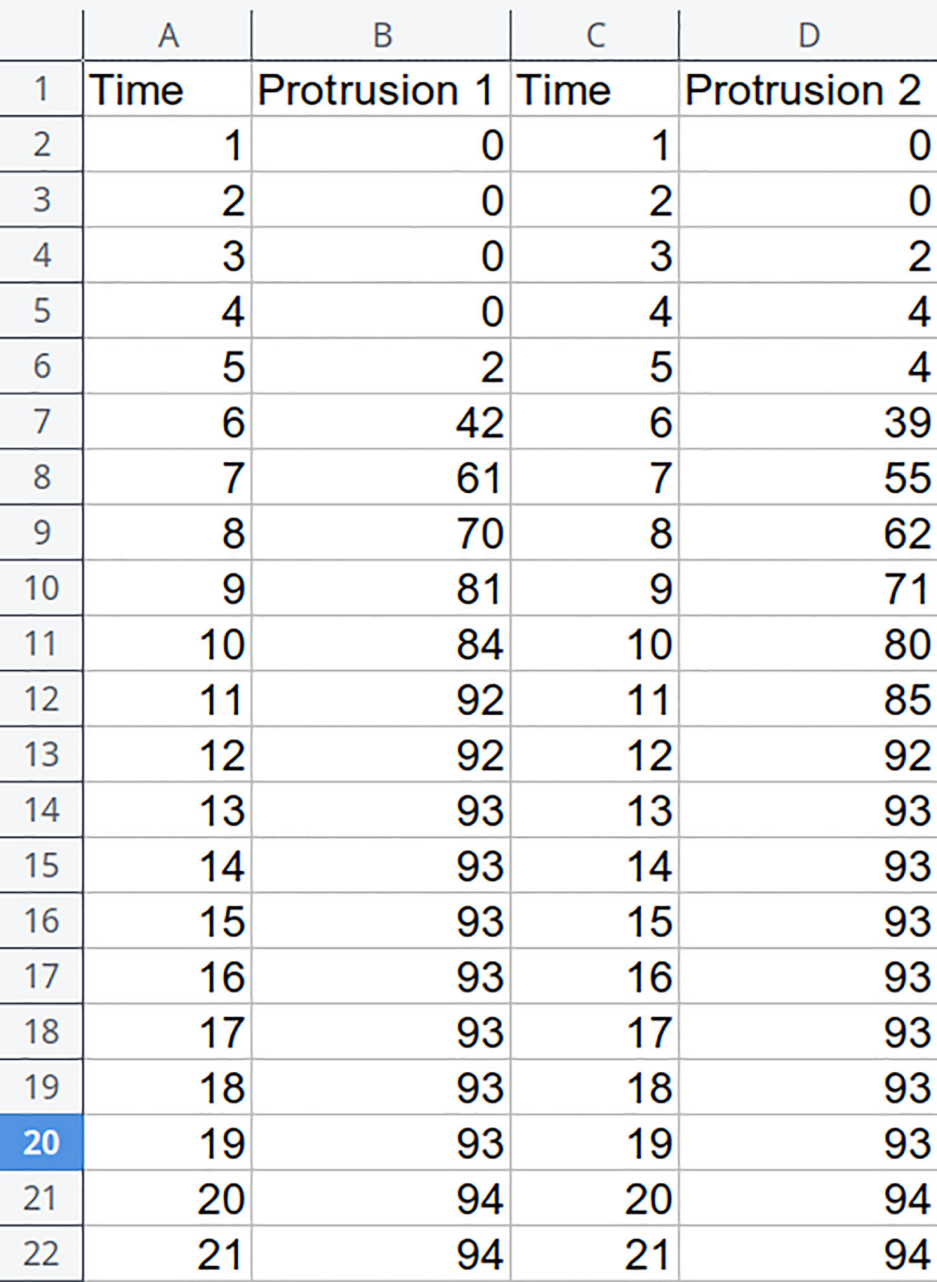
Spreadsheet excerpt illustrating the format supported by POMONA.

POMONA has an intuitive interface, and the data input can be done in a very simple way. **Figure 1** shows a spreadsheet with two measurements, “Protrusion 1” and “Protrusion 2”, with their respective “Time” columns. From this information, POMONA is capable of supplying estimates. It is important to note that datasets with similar nature can also be analyzed by POMONA, such as Normal Seedlings.

#### 3.2.1 Numerical kernel

All linear algebra basic operations used in POMONA were implemented in JavaScript. Although typically not a numerically oriented language, JavaScript has shown that a fairly large number of experiments can have their data fitted for all models in a short time. This can be done on a range of devices from powerful desktop computers to mobile devices.

#### 3.2.2 The longevity assessment module: a case study

In order to illustrate some of the POMONA capabilities, an example is presented of a soybean longevity assessment using four repetitions at different times. For the sake of brevity, the screenshots were taken from its desktop version. The Android app is, for all purposes, identical to the desktop one, with a few idiosyncrasies related to mobile platforms (like automatic screen rotation).

**Figure 2:**
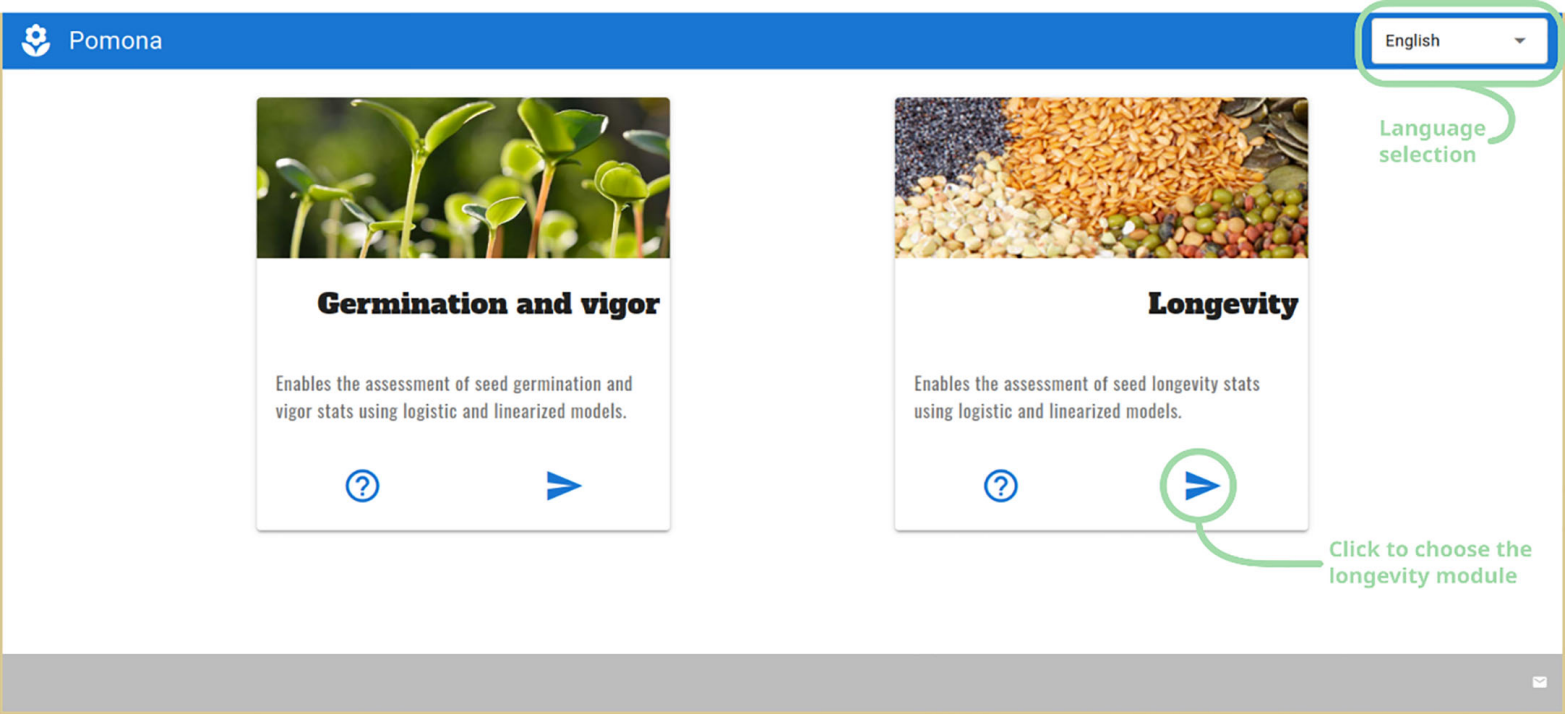
The First POMONA screen, presenting the user with two modules: germination and longevity, along with the language selector at the upper right corner.

When first started, POMONA greets the user with a screen with two choices: germination and longevity assessment modules. On the upper right corner, a dropdown menu brings the language selector. Currently POMONA supports English and Brazilian Portuguese (**Figure 2**).

**Figure 3:**
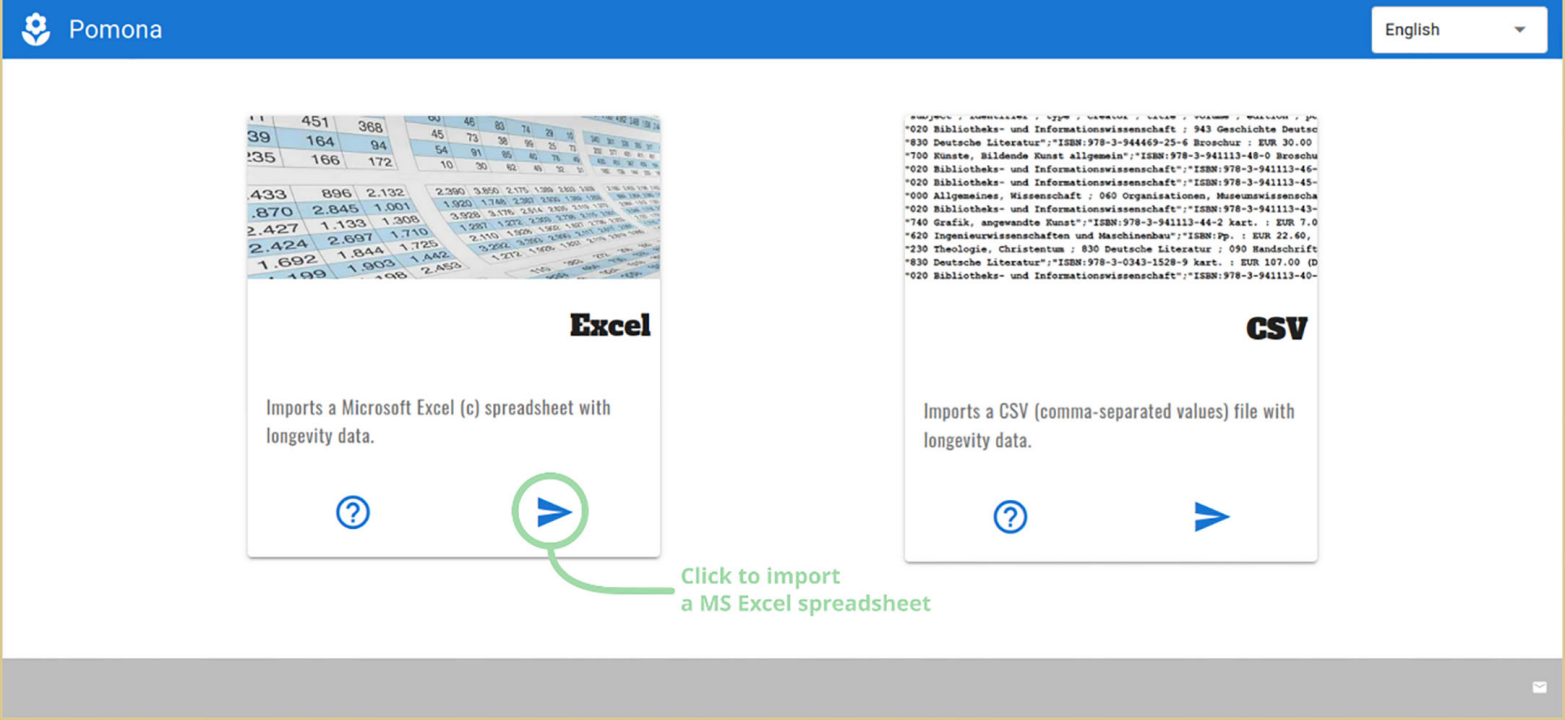
POMONA can import Microsoft Excel or Comma-Separated-Values files.

Once the module is selected, a second screen asks for the kind of file being imported, Microsoft Excel™ or CSV (**Figure 3**). Clicking on the right pointing arrow opens a system-dependent file open dialog: mouse-driven on desktop operating systems, touch-based on Android.

Logistic and linear model fitting is performed with a derivative free analog of the Levenberg-Marquardt method, integrated into POMONA through the levenberg-marquardt JavaScript library, a non-linear fitting library. It provides a set of parameters for fine tuning the method, including damping and error tolerance for the numerical approximation of the Jacobian matrix.

**Figure 4:**
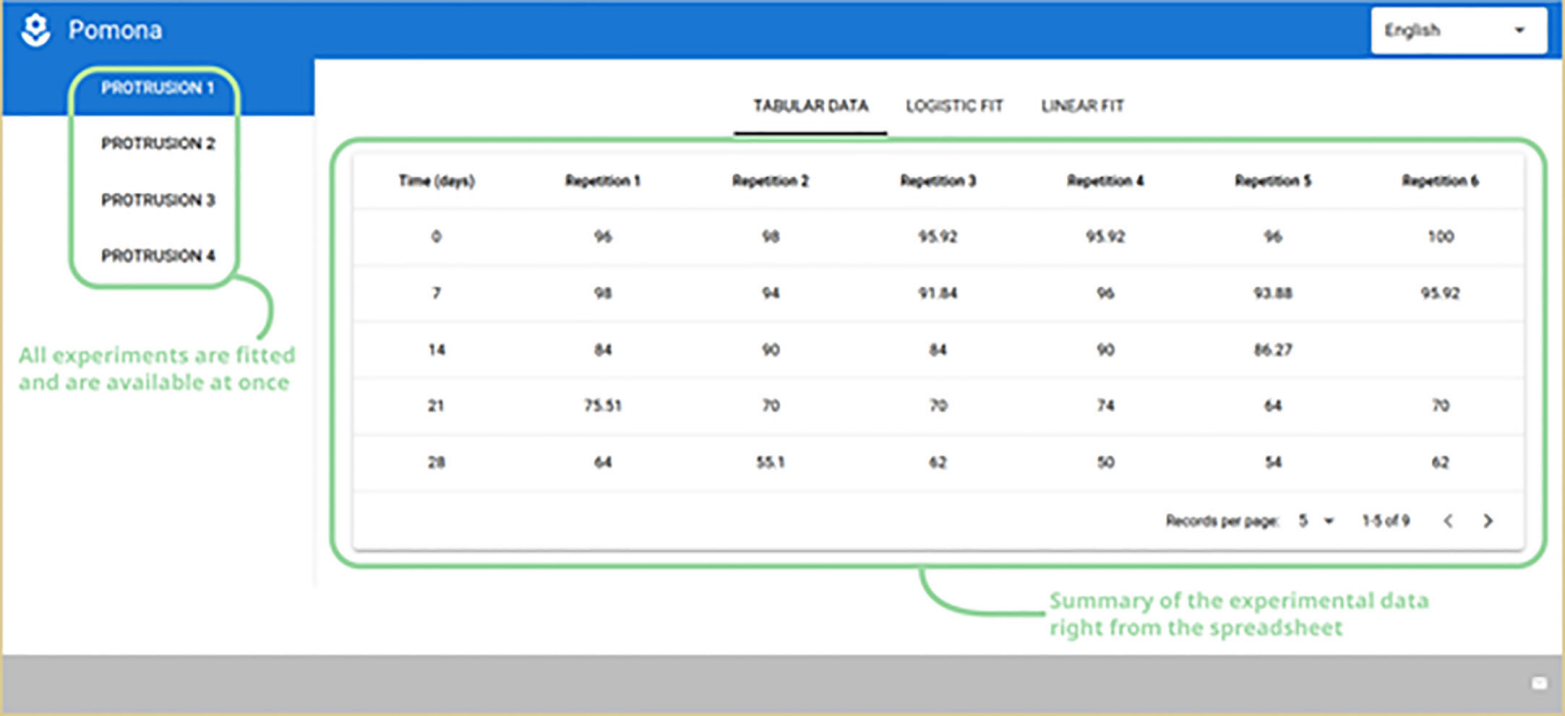
Experiment names on the left and a time-based summary of the experimental data on the right.

From this point on, all relevant logistic and linear models are fitted and made available for the user. A vertical menu is presented on the left with the user given experiment names taken from the spreadsheet. Selecting a different experiment will update the entire interface, including the time-based summary table on the right (**Figure 4**).

**Figure 5:**
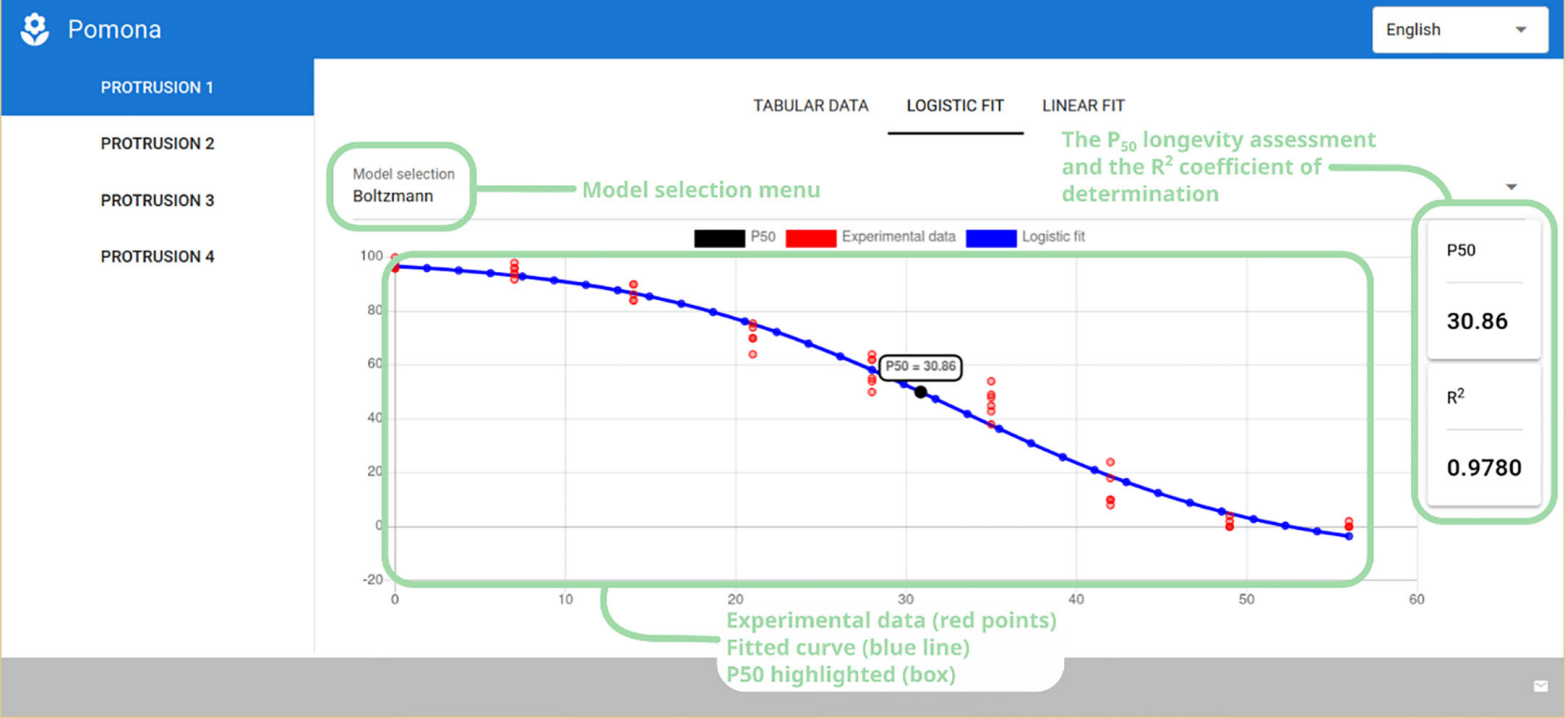
Logistic fit tab showing the model selection menu, P_50_ longevity assessment and coefficient of determination (R^2^), along with the plot of the experimental and fitted data.

Selecting the “Logistic Fit” tab presents the user with a plot of the experimental data (red dots) and the fitted curve (blue line), along with the P_50_ longevity assessment parameter (also highlighted in a box inside the plot), the coefficient of determination (R^2^) and a model selection menu (**Figure 5**). In the case of longevity, the user can choose from the Boltzmann, Gompertz and the Logistic models.

**Figure 6:**
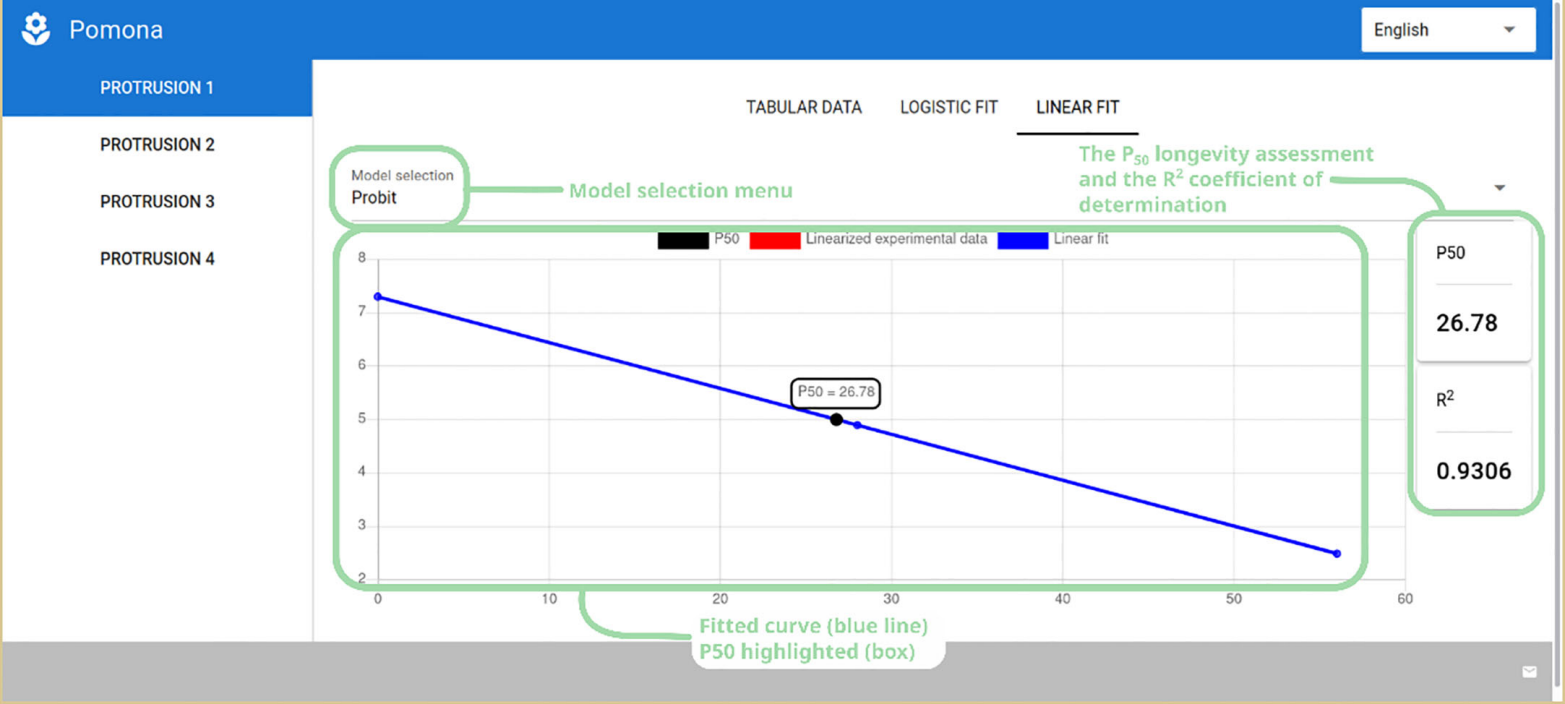
Linear probit fit showing the model selection menu, P_50_ longevity assessment and coefficient of determination (R^2^), along with the plot of the fitted data.

Finally, in the last tab – “Linear Fit” – the user can appreciate the fitted linearized models Probit or Logit. The overall interface is the same as that of the logistic tab, presenting the plot, the model selection menu and the P_50_ and R^2^ values (**Figure 6**).

#### 3.2.3 The germination and vigor assessment module: a case study

**Figure 7:**
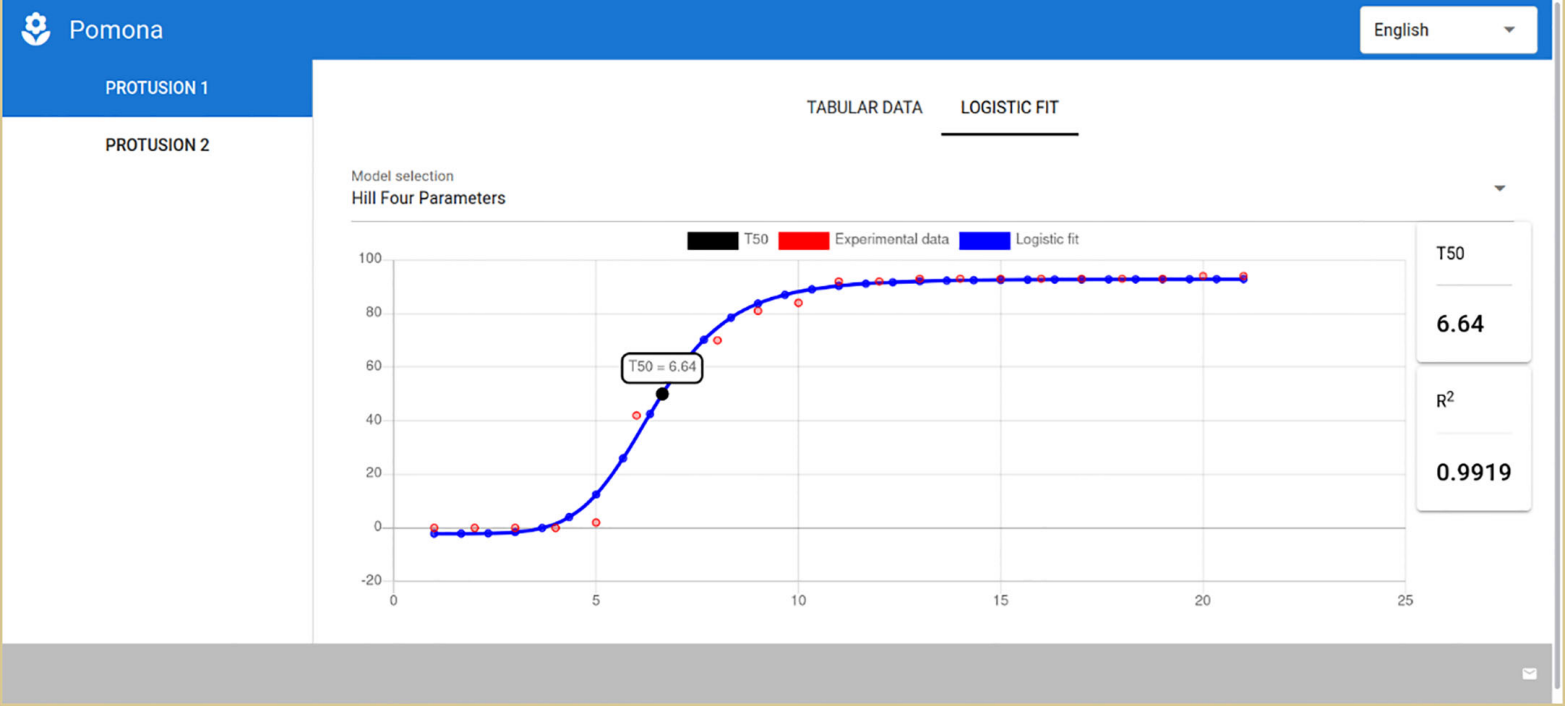
POMONA showing the results for the germination and vigor assessment module.

POMONA has a consistent interface that standardizes the user experience for both modules, meaning that the first few steps for the germination and vigor module are exactly the same as for the longevity one. Therefore, only the final screen with the results of the germination assessment for soybean seeds using the Hill Four-Parameters model (**Figure 7**) is presented. It should be noted that the Gompertz model is also available in this module, and the relevant parameter here is the t_50_.

### 3.3 POMONA on Android devices

**Figure 8:**
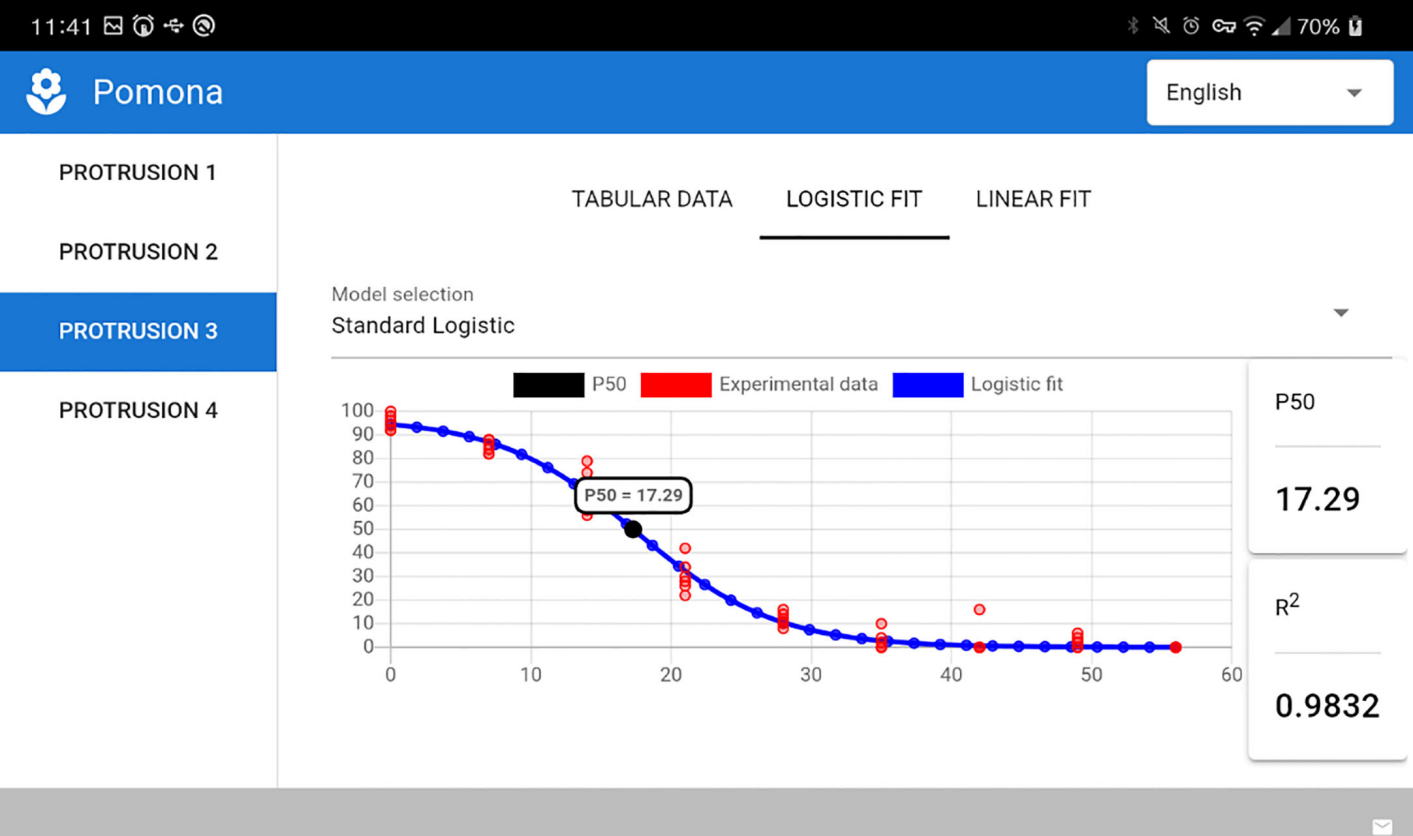
POMONA running on an Android device.

Given its multiplatform nature, POMONA runs as well on mobile devices powered by Android. **Figure 8** presents a screenshot taken on a LG K12 device running Android version 10 (cropped on the right due to screen dimensions).

### 3.4 Modeling germination and longevity as a step to predict P_50_ and t_50_ – POMONA’s validation

To validate POMONA, the logistic model was used for germination and longevity inferences. For both phenomena, parameters estimated by POMONA are between confidence intervals (CI) calculated for the model (**Tables 5**, **6**). Therefore, POMONA is robust to model germination and longevity cumulative over time from a statistical point of view.

**Table 5:** Germination and longevity of soybean (*Glycine max* L.) seed samples of different cultivars estimated from methods for model parameterization.

Cultivar Sample^1^	Parameters^2,#^									
	*LL*≤ $\hat{\beta_{1}}$ ≤*UL*	*LL*≤P_50_(Days)≤*UL*		$\hat{\beta_{1}}$	P_50_ (Days)			t_50_ (h; *R^2^*)		
		Probit	Logit		Probit		Logit	Probit	Logit	
**SAM17**	[60.84; 94.34]	[59.70; 61.24]	[59.26; 62.00]	62	60.47		60.63	54.62(0.43)	54.7(.043)	
**SAM21**	[94.34; 96.66]	[39.68; 39.74]	[39.41; 39.73]	95.5	39.71		39.57	40.75(0.01)	41.1(0.01)	
**SAM02**	[82.10; 82.90]	[7.94; 8.24]	[8.33; 8.57]	82.5	8.09		8.45	34.82(0.74)	34.78(0.73)	
**SAM08**	[70.54; 72.46]	[14.00; 14.24]	[13.06; 13.32]	71.5	14.12		13.19	42.13(0.62)	42.34(0.62)	
**SAM14**	[64.90; 65.10]	[24.64; 26.42]	[21.29; 22.97]	65	25.53		22.13	23.66(0.41)	23.92(0.43)	
**SAM20**	[37.03; 37.17]	[24.75; 27.31]	[26.58; 31.52]	37.1	26.03		29.05	23.02(0.34)	22.59(0.35)	
**SAM07**	[93.15; 93.85]	[40.06; 41.90]	[42.97; 45.63]	93.5	40.98		44.3	46.38(0.70)	46.29(0.70)	
**SAM01**	[84.79; 87.21]	[47.24; 47.48]	[48.10; 48.92]	86	47.36		48.51	14.57(0.34)	14.9(0.34)	
**SAM19**	[49.46; 50.34]	[54.67; 56.07]	[58.92; 60.90]	49.9	55.37		59.91	42.1(0.67)	42.21(0.67)	
**SAM13**	[71.14; 72.86]	[51.55; 51.69]	[57.29; 58.01]	72	51.62		57.65	44.91(0.16)	44.95(0.16)	
**SAM00**	[69.03; 72.97]	[49.45; 52.79]	[53.51; 56.71]	71	51.12		55.11	26.62 (0.77)	26.62(0.78)	
**SAM11**	[75.75; 76.25]	[39.54; 41.02]	[44.89; 46.25]	76	40.28		45.57	38(0.52)	37.99(0.53)	
**SAM05**	[46.22; 51.78]	[56.65; 57.69]	[59.24; 60.80]	49	57.17		60.02	53.78(0.71)	53.46(0.72)	
**SAM12**	[75.07; 76.25]	[39.47; 40.83]	[42.44; 44.06]	79.4	40.15		43.25	19.67(0.36)	20.15(0.37)	
**SAM22**	[46.22; 51.78]	[26.45; 26.99]	[30.03; 30.41]	50.5	26.72		30.22	14.23(0.39)	15.48(0.41)	
**SAM06**	[75.07; 83.73]	[27.52; 28.48]	[27.47; 28.53]	90	28		28	27.79(0.44)	28.15(0.45)	
**SAM18**	[48.66; 52.34]	[17.71; 17.81]	[18.38; 18.56]	59.5	17.76		18.47	28.33(0.27)	27.94(0.26)	
**SAM09**	[84.43; 95.57]	[44.11; 45.11]	[37.89; 39.11]	65.5	44.61		38.5	50.29(0.46)	50.26(0.45)	
**SAM10**	[59.10; 59.90]	[29.06; 30.68]	[29.19; 30.93]	20	29.87		30.06	40.7(0.40)	40.71(0.38)	
**SAM16**	[64.29; 59.90]	[30.10; 30.26]	[30.47; 30.71]	24.5	30.18		30.59	53.33(0.56)	53.28(0.56)	

^1^: Soybean sample code for commercial lots used as biological model; ^2^: Parametrization from logistic models with probit and logit as link functions; $\hat{\beta_{1}}$ : Estimative for slope of logistic model, i.e., maximum germinability; t_50_ (h): median germination time in hours; P_50_ (Days): time in days at which germination characteristically declined to 50%;.^#^: For each functional trait, between LL (lower limit) and UP (upper limit) represents confidence intervals (lower and upper) from model parameters calculated (α = 0:05); whereas single values are estimative of POMONA. R^2^: Coefficient of determination of the model.

The dataset was obtained from stressed seeds by the accelerated aging test (at 75%RH).

**Table 6:** Germination and longevity of corn (*Zea mays* L.) seed samples of different cultivars estimated from methods for model parameterization.

Hybrid Sample^1^	Parameters^2,#^								
	*LL*≤ $\hat{\beta_{1}}$ ≤*UL*	*LL*≤P_50_(Days)≤*UL*		$\hat{\beta_{1}}$	P_50_ (Days)		t_50_ (h; *R^2^*)		
		Probit	Logit		Probit	Logit	Probit		Logit
**MAM03**	[70.16; 70.84]	[12.38; 14.16]	[12.63; 14.33]	70.5	13.27	13.48	50.8(0.99)		50.9(0.99)
**MAM06**	[79.42; 84.58]	[56.67; 63.37]	[58.15; 64.85]	82	60.02	61.5	53.2(0.97)		53.3(0.93)
**MAM05**	[84.18; 86.82]	[15.28; 17.74]	[18.05; 20.47]	85.5	16.51	19.26	52.9(0.96)		53(0.98)
**MAM01**	[67.06; 75.74]	[78.95; 83.39]	[91.52; 95.90]	71.4	81.17	93.71	39.9(0.96)		39.8(0.94)
**MAM04**	[66.08; 68.92]	[98.50; 105.50]	[111.41; 120.25]	67.5	102	115.83	45.4(0.97)		45.1(0.96)

^1^: Corn sample code for commercial lots used as biological model; ^2^: Parametrization from logistic models with probit and logit as link functions; $\hat{\beta_{1}}$ : Estimative for slope of logistic model, i.e., maximum germinability; t_50_ (h): median germination time in hours; P_50_ (Days): time in days at which germination characteristically declined to 50%;.^#^: For each functional trait, between LL (lower limit) and UP (upper limit) represents confidence intervals (lower and upper) from model parameters calculated (α = 0:05); whereas single values are estimative of POMONA. R^2^: Coefficient of determination of the model.

The dataset was obtained from stressed seeds by the cold test.

Estimative of P_50_ for both corn and soybean seeds (all samples) exposed to accelerated aging (75%RH) or cold test from POMONA are in the boundaries of CI for logistic models with probit and logit link functions (**Table 5**). This also demonstrates that the software is viable to predict the longevity parameter.

On the other hand, the t_50_ estimative also proved to be robust in POMONA. In this case, for corn seeds, a great fitting of observed data on both probit and logit link functions showed the robustness for the model to predict viability; whereas for soybean seeds the fitting was minor (**Table 6**). In any case, even though some samples were not fitting for this measurement (SAM01 and SAM09), the parameters of models plotted by POMONA were between the upper and lower confidence intervals calculated (**Table 6**). Therefore, POMONA is also applicable to promote germination analysis from t_50_. It is important to note that t_50_ estimates are also similar to those estimated by Hill’s model, defined for germination process characterization (**Tables 1**, **2**). This reinforces POMONA’s applicability.

## 4 Discussion

POMONA was conceived to be a high-throughput image processing platform of low-cost and free access for scientists and technologists involved with the seed industry. The idea is to give replicability and reproducibility for seed lab results in a friendly multiplatform interface. Thus, a first step was to introduce POMONA as a platform for measurement calculations from models used to explain germination and longevity of seed samples. This was done to overcome a structural barrier of technology transference: difficulties of classical technicians to adopt new technologies (both statistical and computational tools). Other tools were development with this perspective, such as R packages (Lozano-Isla et al., 2019; Silva et al., 2019), spreadsheets (Ranal et al., 2009) and high-throughput scoring as GERMINATOR(Joosen et al., 2010) and SeedGem(Colmer et al., 2020). These solutions have limitations such as the need to know a programing language, or model fitting restrictions. Thus, POMONA is introduced *a priori* as a complementary tool for indexes up to now not calculated by a free-access seed science platform. To support this, our findings were based on two structures: experimental and theoretical to support POMONA’s applicability.

First, pre-testing was observed to be effective to provide at least three groups of physiological quality in seed-seedling transition for both biological models. That was possible mainly due to seedling vigor status and a deep study on kinetics, time and indexes related to regularity of germination events over time. This is not new, and for the last two decades has been defended by seed scientists such as Ranal and de Santana (2006), who strived to provide robust and pure measurements to analyze seed-seedling transition. Here, the fitting curve of GERMINATOR was used as part of the physiological characterization, but classical measurements from Ranal et al. (2009) were also used. Taking this into account, it was observed that corn seeds were more predictable than soybean seeds regarding germination and normal seedling patterns. This was expected and it occurs due to anatomical peculiarities for each biological model. Soybean seeds are a ‘true seed’, whereas corn seeds are caryopses (Bewley et al., 2013 sense). A consequence of this is a higher sensitivity of soybean in early steps of germination which hinders estimative, compared to what is observed in corn seeds (see Ribeiro-Oliveira et al., 2020). Therefore, model fitting should be more accurate for seed-seedling transition of corn seeds than soybean seeds. It is important to note that from a seed technology point of view, only seed samples with high seedling development patterns can be used for trade (ISTA, 2019). Here the main concern is not with causes of differences from abnormal seedlings, poor seed viability or seedling development, including response due to the genotype factor. For the research these measurements were only used as a way to prove physiological differences. From a statistical and physiological point of view, using sample groups with an intermediate and low capacity of seed-seedling transition enables more robust inferences (Ribeiro-Oliveira and Ranal, 2016; Ribeiro-Oliveira et al., 2016; Ribeiro-Oliveira and Ranal, 2018; Ribeiro-Oliveira et al., 2020), and this was considered in order to keep all samples of each biological model. From this point, it was observed that POMONA is applicable to model germination (viability), vigor and longevity, especially when compared to Hill’s model.

Recent discussions of seed scientists have been provided regarding the robustness of models associated with the Normal distribution in relation to generalized linear models (GLMs, see Carvalho et al., 2018; Ribeiro-Oliveira et al., 2018). This perspective pointed out that GLMs are robust due to a more flexible model fit, and non-dependent of Normal distribution (Mcnair et al., 2012; dos Santos et al., 2019; de Faria et al., 2020; Amorim et al., 2021). Therefore, logistic models with different link functions were considered a new tool to measure the viability (t_50_) and longevity (P_50_) of commercial seed lots of cultivated species, such as corn and soybean (dos Santos et al., 2019; de Faria et al., 2020). This idea was used here to validate POMONA. In all cases studied, POMONA parametrization was within the confidence intervals provided for model parameters. In addition, from the Normal distribution, parameters of link functions and Hill’s model had similar estimates. Therefore, the software proved to be robust in a statistical sense.

What is new in all of this, is the way POMONA has been developed. It has a core that includes I/O routines, numerical methods and statistical models, is implemented in JavaScript, a mature language powering much of the modern internet and many mobile applications, while its graphical user interface uses Quasar (Vue.js Guide, https://vuejs.org/v2/guide), an enterprise-grade, high-performance framework for multiplatform application development. Quasar seamlessly integrates a single page application (SPA) written in JavaScript with both the Electron framework (Electron Framework, https://electronjs.org) and the Capacitor runtime (ElementUI, A Desktop UI Library, https://element.eleme.io/#/en-US). The Electron integration turns POMONA into a native desktop application on Microsoft Windows (version 10, or better) or any modern GNU/Linux distribution. On the other hand, Capacitor brings POMONA to Android devices (version 7, or better). Being developed in JavaScript, POMONA can be hosted on an internet server as a typical webpage. POMONA can read and parse Microsoft Excel™ spreadsheets or comma-separated-values (CSV) files. In both cases, each repetition must comprise two columns, one for the discretized time, another for the germination percentage or radicle protrusion. Several measurements can be modelled simultaneously, each one of them using two consecutive columns. Note that the time discretization does not need to be homogeneous among experiments. Therefore, POMONA has shown be applicable in the seed industry. It is important to highlight that the implementations regarding image capture and processing are the next step for POMONA and, in this case, an artificial intelligence algorithm is being development. In any case, here POMONA is introduced as a new and viable multiplatform tool for seed viability and longevity analysis.

## Data availability statement

The raw data supporting the conclusions of this article will be made available by the authors, without undue reservation.

## Author contributions

RC, JR-O, ES, and MS devised the research and the main conceptual ideas. AS processed the data record together with RF. AS, RF and MS were involved in data analysis. RC, JR-O and ES drafted the manuscript and designed the figures. All authors contributed to the article and approved the submitted version.

## References

[B1] Amorim D. J. Pereira Dos Santos A. R. Nunes da Piedade G. Quelvia de Faria R. Amaral da Silva E. A. Pereira Sartori M. M. (2021). The use of the generalized linear model to assess the speed and uniformity of germination of corn and soybean seeds. Agronomy 11, 588. doi: 10.3390/AGRONOMY11030588

[B2] Barboza da Silva C. Bianchini V. d. J.M. Medeiros A.D. d. Moraes M.H.D. d. Marassi A. G. Tannús A. (2021). A novel approach for jatropha curcas seed health analysis based on multispectral and resonance imaging techniques. Ind. Crops Prod. 161. doi: 10.1016/j.indcrop.2020.113186

[B3] Barros S. R. B. Dias M. C. L. L. Cicero S. M. Krzyzanowski F. C. (1999). “Teste frio,” in Vigor de sementes: conceitos e testes. Eds. Krzyzanowski F. C. Vieira R. D. França-Neto J. B. (Londrina, PR, Brazil: ABRATES), 1–15.

[B4] Batista T. B. Mastrangelo C. B. de Medeiros A. D. Petronilio A. C. P. Fonseca de Oliveira G. R. dos Santos I. L. . (2022). A reliable method to recognize soybean seed maturation stages based on autofluorescence-spectral imaging combined with machine learning algorithms. Front. Plant Sci. 13, 2037. doi: 10.3389/FPLS.2022.914287/BIBTEX PMC9237540 35774807

[B5] Benos L. Tagarakis A. C. Dolias G. Berruto R. Kateris D. Bochtis D. (2021). Machine learning in agriculture: a comprehensive updated review. Sensors 21, 3758. doi: 10.3390/S21113758 34071553 PMC8198852

[B6] Bewley J. D. Bradford K. J. Hilhorst H. W. M. Nonogaki H. (2013). Seeds (New York, NY: Springer New York). doi: 10.1007/978-1-4614-4693-4

[B7] Carvalho F. J. de Santana D. G. de Araújo L. B. (2018). Why analyze germination experiments using generalized linear models? J. Seed Sci. 40, 281–287. doi: 10.1590/2317-1545V40N3185259

[B8] Colmer J. O’Neill C. M. Wells R. Bostrom A. Reynolds D. Websdale D. . (2020). SeedGerm: a cost-effective phenotyping platform for automated seed imaging and machine-learning based phenotypic analysis of crop seed germination. New Phytol. 228, 778–793. doi: 10.1111/NPH.16736 32533857

[B9] Coolbear P. Francis A. Grierson D. (1984). The effect of low temperature pre-sowing treatment on the germination performance and membrane integrity of artificially aged tomato seeds. J. Exp. Bot. 35, 1609–1617. doi: 10.1093/JXB/35.11.1609

[B10] de Faria R. Q. dos Santos A. R. P. Amorim D. J. Cantão R. F. da Silva E. A. A. Sartori M. M. P. (2020). Probit or logit? which is the better model to predict the longevity of seeds? Seed Sci. Res. 30, 49–58. doi: 10.1017/S0960258520000136

[B11] de Medeiros A. D. da Silva L. J. Ribeiro J. P. O. Ferreira K. C. Rosas J. T. F. Santos A. A. . (2020). Machine learning for seed quality classification: an advanced approach using merger data from FT-NIR spectroscopy and x-ray imaging. Sens. (Switzerland) 20, 1–12. doi: 10.3390/s20154319 PMC7435829 32756355

[B12] dos Santos A. R. P. de Faria R. Q. Amorim D. J. Giandoni V. C. R. da Silva E. A. A. Sartori M. M. P. (2019). Cauchy, Cauchy–Santos–Sartori–Faria, logit, and probit functions for estimating seed longevity in soybean. Agron. J. 111, 2929–2939. doi: 10.2134/AGRONJ2018.11.0700

[B13] Ducournau S. Feutry A. Plainchault P. Revollon P. Vigouroux B. Wagner M. H. (2005). Using computer vision to monitor germination time course of sunflower (Helianthus annuus l.) seeds. Seed Sci. Technol. 33, 329–340. doi: 10.15258/sst.2005.33.2.06

[B14] ECMA International (2017). Standard ECMA-262, ECMAScript 2017, language specification, 8th ed. (New York: ECMA International).

[B15] El-Kassaby Y. A. Moss I. Kolotelo D. Stoehr M. (2008). Seed germination: mathematical representation and parameters extraction. For. Sci. 54, 220–227. doi: 10.1093/FORESTSCIENCE/54.2.220

[B16] Ellis R. H. Roberts E. H. (1980). The influence of temperature and moisture on seed viability period in barley (Hordeum distichum l.). Ann. Bot. 45, 31–37. doi: 10.1093/OXFORDJOURNALS.AOB.A085798

[B17] Galletti P. A. Carvalho M. E. A. Hirai W. Y. Brancaglioni V. A. Arthur V. Barboza da Silva C. (2020). Integrating optical imaging tools for rapid and non-invasive characterization of seed quality: tomato (Solanum lycopersicum l.) and carrot (Daucus carota l.) as study cases. Front. Plant Sci. 11, 577851. doi: 10.3389/fpls.2020.577851 PMC7779677 33408727

[B18] Horler R. S. P. Turner A. S. Fretter P. Ambrose M. (2018). SeedStor: a germplasm information management system and public database Plant Cell Physiol. doi: 10.1093/pcp/pcx195 PMC5914401 29228298

[B19] ISTA (2019). International rules for seed testing 2019. Int. Rules Seed Testing. doi: 10.15258/istarules.2019.10

[B20] Joosen R. V. L. Kodde J. Willems L. A. J. Ligterink W. van der Plas L. H. W. Hilhorst H. W. M. (2010). Germinator: a software package for high-throughput scoring and curve fitting of arabidopsis seed germination. Plant J. 62, 148–159. doi: 10.1111/J.1365-313X.2009.04116.X 20042024

[B21] Katalin M. Powell M. A. Zecchinelli M. R. (2009). Session 4 THE IMPORTANCE OF QUALITY SEED IN AGRICULTURE What is seed quality and how to measure it? The influence of seed quality on crop productivity.

[B22] Labouriau L. G. A. (1970). On the physiology of seed germination in vicia graminea I. Acad. Bras. Cienc. 42, 235–262.

[B23] Labouriau L. G. A. (1983). Germinação de sementes (Caracas, Venezuela: Secretaria Geral da Organização dos Estados Americanos).

[B24] Ligterink W. Hilhorst H. W. M. (2017), 57–72. doi: 10.1007/978-1-4939-6469-7_7 27864758

[B25] Lozano-Isla F. Benites-Alfaro O. E. Pompelli M. F. (2019). GerminaR: an r package for germination analysis with the interactive web application “GerminaQuant for r”. Ecol. Res. 34, 339–346. doi: 10.1111/1440-1703.1275

[B26] Mcnair J. N. Sunkara A. Frobish D. (2012). How to analyse seed germination data using statistical time-to-event analysis: non-parametric and semi-parametric methods Seed Sci. Res.22 (2), 77–95. doi: 10.1017/S0960258511000547

[B27] Merieux N. Cordier P. Wagner M.-H. Ducournau S. Aligon S. Job D. . (2021). ScreenSeed as a novel high throughput seed germination phenotyping method. Sci. Rep. 11, 1404. doi: 10.1038/s41598-020-79115-2 33446694 PMC7809209

[B28] Oliveira G. R. F. Mastrangelo C. B. Hirai W. Y. Batista T. B. Sudki J. M. Petronilio A. C. P. . (2022). An approach using emerging optical technologies and artificial intelligence brings new markers to evaluate peanut seed quality. Front. Plant Sci. 13. doi: 10.3389/FPLS.2022.849986 PMC9048030 35498679

[B29] Pavicic M. Wang F. Mouhu K. Himanen K. (2019). High throughput *in vitro* seed germination screen identified new ABA responsive RING-type ubiquitin E3 ligases in arabidopsis thaliana. Plant Cell Tissue Organ Cult. 139, 563–575. doi: 10.1007/S11240-019-01700-9/FIGURES/4

[B30] Ranal M. A. de Santana D. G. (2006). How and why to measure the germination process? Braz. J. Bot. 29, 1–11. doi: 10.1590/S0100-84042006000100002

[B31] Ranal M. A. Santana D.G. d. Ferreira W. R. Mendes-Rodrigues C. (2009). Calculating germination measurements and organizing spreadsheets. Rev. Bras. Botânica 32. doi: 10.1590/s0100-84042009000400022

[B32] Rejeb A. Rejeb K. Zailani S. Keogh J. G. Appolloni A. (2022). Examining the interplay between artificial intelligence and the agri-food industry. Artif. Intell. Agric. 6, 111–128. doi: 10.1016/J.AIIA.2022.08.002

[B33] Ribeiro-Oliveira J. P. Ranal M. A. (2014). Sementes florestais brasileiras: início precário, presente inebriante e o futuro, promissor? Ciencia Florestal. 24 (3), 771–784. doi: 10.5902/1980509815738

[B34] Ribeiro-Oliveira J. P. Ranal M. A. (2016). Sample size in studies on the germination process. Botany 94, 103–115. doi: 10.1139/cjb-2015-0161

[B35] Ribeiro-Oliveira J. P. Ranal M. A. (2018). Sample size and water dynamics on germinating diaspores: the first step for physiological and molecular studies on the germination process. Plant Biosyst. 152, 840–847. doi: 10.1080/11263504.2017.1353551

[B36] Ribeiro-Oliveira J. P. Ranal M. A. Boselli M. A. (2020). Water dynamics on germinating diaspores: physiological perspectives from biophysical measurements. Plant Phenomics 2020, 1–16. doi: 10.34133/2020/5196176 PMC7869936 33575666

[B37] Ribeiro-Oliveira J. P. Ranal M. A. Garcia De Santana D. Pereira L. A. (2016). Sufficient sample size to study seed germination. Aust. J. Bot. 64. doi: 10.1071/BT15254

[B38] Ribeiro-Oliveira J. P. Santana D.G. d. Pereira V. J. Santos C.M.d. (2018). Data transformation: an underestimated tool by inappropriate use. Acta Sci. Agron. 40, 1–11. doi: 10.4025/actasciagron.v40i1.35300

[B39] Silva L. J. D. Dantas De Medeiros A. Morbeck A. Oliveira S. (2019). SeedCalc, a new automated r software tool for germination and seedling length data processing. J. Seed Sci. 41, 250–257. doi: 10.1590/2317-1545V42N2217267

[B40] USDA (2022). “Oilseeds: world markets and trade,” in Global oilseed consumption continues to grow despite slowing trade and production. (Madision, Wisconsin, USA: USDA). doi: 10.1016/S1097-8690(11)70006-3

